# Evaluation of methods for extraction of the volitional EMG in dynamic hybrid muscle activation

**DOI:** 10.1186/1743-0003-3-27

**Published:** 2006-11-23

**Authors:** Eran Langzam, Eli Isakov, Joseph Mizrahi

**Affiliations:** 1 Department of Biomedical Engineering – Technion, Israel Institute of Technology, Haifa, Israel; 2 Loewenstein Rehabilitation Center, Raanana, Israel

## Abstract

**Background:**

Hybrid muscle activation is a modality used for muscle force enhancement, in which muscle contraction is generated from two different excitation sources: volitional and external, by means of electrical stimulation (ES). Under hybrid activation, the overall EMG signal is the combination of the volitional and ES-induced components. In this study, we developed a computational scheme to extract the volitional EMG envelope from the overall dynamic EMG signal, to serve as an input signal for control purposes, and for evaluation of muscle forces.

**Methods:**

A "synthetic" database was created from *in-vivo *experiments on the Tibialis Anterior of the right foot to emulate hybrid EMG signals, including the volitional and induced components. The database was used to evaluate the results obtained from six signal processing schemes, including seven different modules for filtration, rectification and ES component removal. The schemes differed from each other by their module combinations, as follows: blocking window only, comb filter only, blocking window and comb filter, blocking window and peak envelope, comb filter and peak envelope and, finally, blocking window, comb filter and peak envelope.

**Results and conclusion:**

The results showed that the scheme including all the modules led to an excellent approximation of the volitional EMG envelope, as extracted from the hybrid signal, and underlined the importance of the artifact blocking window module in the process.

The results of this work have direct implications on the development of hybrid muscle activation rehabilitation systems for the enhancement of weakened muscles.

## Background

Electromyography (EMG) is an important tool in the fields of biomechanics and kinesiology. In the time domain, the envelope of the rectified EMG signal is commonly used for several applications including: force estimator [1], muscle activity indicator [2], fatigue indicator [3], and more recently as a bio-control signal (e.g: [4-9]).

The term Hybrid muscle activation, coined by the present authors [10,11] is a modality where muscle contraction is generated from two different excitation sources, volitional and external electrical stimulation (ES). This modality has been described in previous works, usually for the enhancement of deficient muscles [5,6,8,11-14]. In hybrid activation, the overall EMG signal is the combination of the volitional and the induced components.

A typical muscle response to ES includes the stimulus artifact, in the form of a spike which immediately follows the electrical stimulus; and an M-wave response which appears afterwards. Due to the fact that the spike's major effect lasts just a few milliseconds, it can be eliminated by using various methods, such as time-windowing [15]. On the other-hand, the M-wave response spreads over most of the inter-pulse time and has a characteristic and repetitive general shape whose specific features depend on factors such as stimulus intensity, shape and polarity.

Current knowledge on the mode of interaction between the volitional and the ES components of the EMG is ambiguous. Early works assumed that in the hybrid EMG signal those two components are simply added up, reflecting the muscle electrical activity when volitional and ES activations take place simultaneously [12]. Recently, however, we were able to show that this assumption is not accurate [10,11].

Extraction of the volitional component from the overall EMG signal thus requires the elimination of the ES component. This is feasible by using hardware and/or software techniques.

Hardware solutions were suggested to suppress the stimulus artifact by using a signal blocking window [4-6,8,11-19]. Software solutions were used for both stimulus artifact and M-wave elimination [6-8,12,20-23] and were achieved by a variety of signal processing filters, including the comb-filter [6,12], wavelets [22,23], adaptive filters [16], or Gram-Schmidt filters [7].

Despite the many methods for the elimination of the ES-induced component, providing the volitional only EMG component, most of them do not provide an evaluation of the accuracy of the extraction procedure i.e., how well the procedure resolves a hybrid signal into its components. The few reports that did so, based their evaluation on steady muscle contraction analysis, and involved mathematical procedures that are difficult to implement [7,16].

In this paper we compare methods for the accurate extraction of the volitional EMG envelope out of the raw dynamic EMG signal. A synthesized, well-defined and known EMG signal, served for the development and validation of the preferred method. Hybrid activation of the muscle was represented by the combined contributions of the volitional and ES induced muscle contractions under dynamic conditions of muscle activation, simulating *in vivo *gait-like contractions of the Tibialis Anterior (TA) muscle. The method of choice was reached by comparing the scoring results from several processing algorithms.

## Methodology

### Experimental procedure

#### Subjects

Five subjects (see details in Table 1) participated in the study. The subjects had an average (SD) age of 28.6 (5.4) years, height of 1.77 (0.13) m, and mass of 66.2 (12.7) kg. All subjects were in an excellent state of health, with no history of muscle weakness, neurological disease or drug therapy. The experiment was approved by the local ethical committee and each subject provided informal consent according to the local ethical committee's guidelines.

**Table 1:** Details of the subjects taking part in this study and their respective testing protocol parameters

Subject	Sex	Age [years]	Height [m]	Mass [Kg]	Volitional Torque levels [%MVC]	ES intensities [mAmp]
1	M	33	1.95	83.0	5, 10, 15, 20, 25	3, 5, 7, 10, 12, 15, 20
2	M	35	1.70	60.0	5, 10, 15, 20, 25	3, 5, 7, 10, 12, 15
3	F	25	1.62	51.0	5, 10, 15, 20, 25	3, 5, 7, 10, 12
4	M	22	1.84	75.0	5, 10, 15, 20, 25	3, 5, 7, 10, 12, 15
5	M	28	1.75	62.0	5, 10, 15, 20, 25	3, 5, 7, 10, 12, 15, 20

Avg.		28.6 (5.4)	1.77 (0.13)	66.2 (12.7)		

#### EMG & mechanical measurements

**Figure 1:**
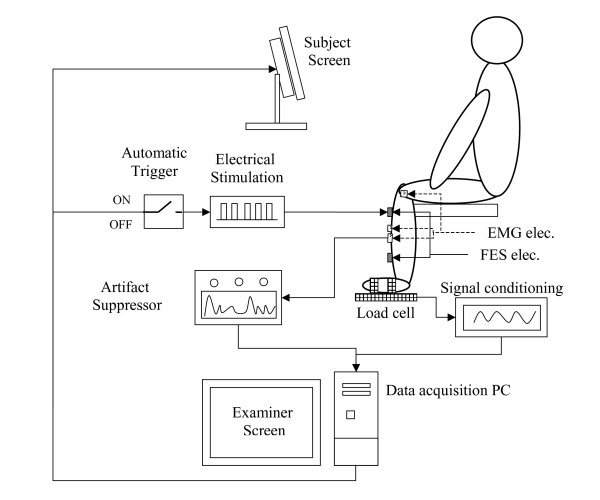
Experimental set-up used for data acquisition.

On-line readings of the ankle torque and EMG of the right TA muscle were taken during each of the experimental trials. The isometric torque was monitored in the seated position by means of a load cell connected on one side to a holding fixture and on the other side to a plate which served as a foot rest and to which the foot was strapped (Fig. 1). The torque due to gravitation was compensated for from the load cell readings while the foot rested on the plate. At this stage, the subject was instructed to relax his leg muscles. Relaxation was confirmed from the unnoticed EMG signal obtained from the monitored muscles. During the test, the ankle, knee and hip angles were set at 90°.

EMG was monitored using three surface SKINTACT^® ^Ag-AgCl circular electrodes (contact diameter of 1.5 cm, external diameter of 5 cm): two active electrodes were located on the muscle belly along its longitudinal axis at mid-distance between the ES stimulation electrodes (see below), the distance between the electrodes was 6 cm center-to center [5,12,15]. The third electrode was a ground electrode and was placed on the bony medial epicondyle area of the knee femur. Before attachment of the electrodes the skin surface was cleaned and rubbed until the electrical impedance between each pair of electrodes was smaller than 5 KΩ. The three electrodes were connected to a specially designed 10 kHz bandwidth DC amplifier with stimulus artifact suppression [15].

The artifact suppressor [15] consists of a sample-and-hold amplifier. The amplifier board is designed to synchronize with the stimulation pulses and to hold the output for a period of 2 milliseconds from the stimulation-pulse onset.

The torque and EMG signals were sampled at a sampling rate of 1 KHz.

Transcutaneous stimulation was delivered to the muscle using two surface rectangular electrodes (5 × 5 cm): one placed over the TA motor point, and the second, 20 cm distally to the first one (center-to-center). The stimulation parameters were controlled through a PC.

Both electrode-sets distances were slightly modified (if necessary), to obtain an overall optimal performance of both EMG, and ES system.

#### MVC measurement

Before any experimental activity, measurement of the Maximum Voluntary Contraction (*MVC*) was carried out. The subject was asked to maximally and isometrically contract his right TA muscle for 5 s. A time window of 0.5 s from the maximal contraction plateau was taken to calculate the mean torque and mean envelope of the rectified EMG signals. Three trials were made, with 5 min of resting time between them, and the average was taken to represent *MVC *for normalization of the torque and EMG.

#### Volitional torque/EMG signals

**Figure 2:**
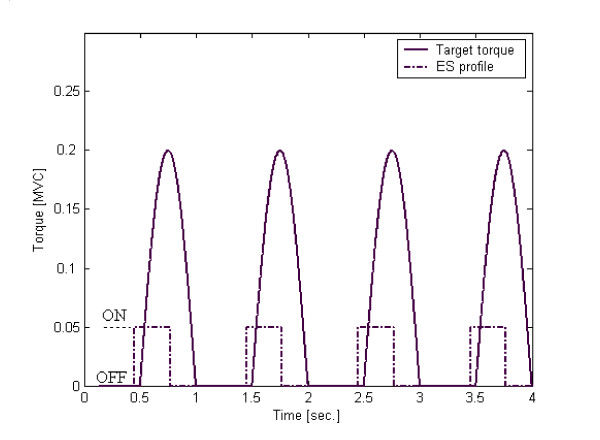
Target torque (solid line) and ES profile (dash-dot line, ON/OFF modes) used to synthesize the EMG database. The target torque amplitude varied from 0.05 to 0.25 *MVC*.

For the acquisition of the volitional EMG signals the subjects were requested to volitionally track a visually displayed torque-time profile by isometrically activating the Tibialis Anterior (TA) muscle through the application of a dorsi-flexion torque at the ankle. The general features of the target torque (*T*_*target*_), mimic the TA torque activity observed during the late swing phase of human gait (Fig. 2) [24]; inability to provide this torque is directly related to biomechanical problems such as drop foot.

The session included 5 trials, each of 15 s duration, with five min interval time between them for resting. The trials differed from each other by their amplitudes, which were varied between 0.05 to 0.25 *MVC*, at increasing steps of 0.05 *MVC*, and providing altogether five levels of volitional activity. The 0.25 *MVC *limit was chosen because this is the typical range of the TA volitional torque during swing [2]. Table 1 describes the specific volitional torques levels for each subject.

#### Induced EMG signals

For acquisition of the EMG signals from the electrically induced contractions, the subject was instructed to stop any volitional activation while his muscle was subjected to the dynamic stimulation profile depicted in Fig. 2. Justification of this profile is based on the role of ES, which was intended to serve for enhancement of the volitional activation [11]. Thus it was necessary to set the ES signal in concert with the target torque signal from the following aspects: (a) duration of the ES signal; (b) synchronization between the two signals. ES was applied by a constant current, electrical stimulator providing 0.3 sec stimulation trains with mono-phasic rectangular pulses of 100 μs duration, and frequency of 20 Hz [25].

An automatic operation signal was used to dynamically turn the stimulator on and off. The ES activation session was performed five min after completion of the volitional session. The trials differed from one another by the stimulation intensity, which was set to cover induced torque amplitudes, ranging between 0.05 to 0.15 *MVC*; the 0.15 MVC limitation was determined as the highest intensity in which the subjects felt comfortable. For the subjects taking part in this study 5 trials or more were necessary to cover this range. Also here, the time length of each trial was 15 s and the resting time between successive trials was 5 min. Table 1 describes the ES intensities that were used to stimulate each subject.

### EMG database

A database of the EMG signal was set to gain information on the partition between the volitional and ES-induced components in the overall EMG signal. The database was produced from *in-vivo *experimental data. A basic signal in the database was the simple summation of the EMG signals that were acquired from volitional excitation only and ES-excitation only muscle tests. The entire database normally included 25 or more synthesized EMG signals, which covered the above described combinations volitional and ES trials.

### Processing of the EMG signal

Several processing schemes of the EMG signal were used to extract the volitional EMG envelope from the raw EMG signal, which included both of the volitional and the ES components. Each scheme comprised several stages, which can be divided into two block groups: rigid blocks whose inclusions are mandatory in the scheme, and flexible blocks, whose inclusions in the scheme are optional. The effect of the flexible blocks was tested by their progressive inclusion into the processing scheme.

**Figure 3:**
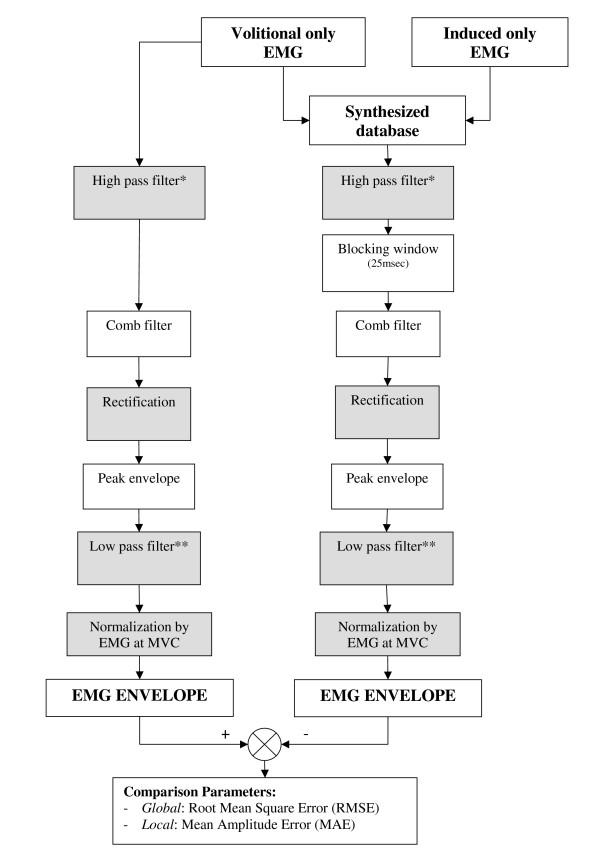
A Processing scheme of the EMG signal. The grey blocks designate "Rigid" processing modules and their inclusion was mandatory in the scheme. The white blocks designate "Flexible" processing modules whose inclusions in the scheme are optional, and they were inserted into and out of the process in various combinations. * Butterworth, 4^th ^order, with cut-offfrequency of 15 Hz. ** Butterworth, 4^th ^order, with cut-off frequency of 5 Hz.

The complete processing scheme thus included the following steps (Fig. 3):

(a) The raw EMG was high-passed filtered (Butterworth HPF, order 4, cutoff frequency 15 Hz);

(b) For the ES-induced signal only, ES artifacts were removed from the EMG using a blocking window of 25 ms. The blocking window started at a beginning of the stimulus artifact pulse and ended 25 msec later [12]: *EMG*(*t*_*stimulus*_) = 0, *EMG*(*t*_*stimulus *_+ 1 *msec*) = 0,..., *EMG*(*t*_*stimulus *_+ 25 *msec*) = 0

(c) Comb-filter was used [12] to retain the volitional only component of the signal:

y(n)=x(n)−x(n−NStim)2     (1)
 MathType@MTEF@5@5@+=feaafiart1ev1aaatCvAUfKttLearuWrP9MDH5MBPbIqV92AaeXatLxBI9gBaebbnrfifHhDYfgasaacH8akY=wiFfYdH8Gipec8Eeeu0xXdbba9frFj0=OqFfea0dXdd9vqai=hGuQ8kuc9pgc9s8qqaq=dirpe0xb9q8qiLsFr0=vr0=vr0dc8meaabaqaciaacaGaaeqabaqabeGadaaakeaacqWG5bqEcqGGOaakcqWGUbGBcqGGPaqkcqGH9aqpdaWcaaqaaiabdIha4jabcIcaOiabd6gaUjabcMcaPiabgkHiTiabdIha4jabcIcaOiabd6gaUjabgkHiTiabd6eaonaaBaaaleaacqWGtbWucqWG0baDcqWGPbqAcqWGTbqBaeqaaOGaeiykaKcabaWaaOaaaeaacqaIYaGmaSqabaaaaOGaaCzcaiaaxMaadaqadaqaaiabigdaXaGaayjkaiaawMcaaaaa@48DB@

Where *x*(*n*) is the n^th ^sample of the actual EMG signal, *N*_*stim *_is the stimulus duration expressed in number of samples and *y*(*n*) is the filtered EMG signal.

(d) The volitional EMG was rectified: *EMG*_*Rectified_i *_= *abs*(*EMG*_*volitional_i*_)

(e) The signal envelope was obtained by connecting the rectified signal peaks by straight lines.

(f) The envelope was low-pass filtered, introducing a smooth envelope profile (Butterworth LPF, order 4, cutoff frequency 5 Hz), and

(g) The smoothed envelope was normalized to *EMG*_*MVC*_, as previously defined: *EMG*_*Normalized *_*= EMG*_*envelope*_*/EMG*_*MVC*_.

The "rigid" modules thus included steps (a), (d), (f), and (g); and the "flexible" modules included steps (b), (c), and (e). Six different schemes, covering all possible combinations of "flexible" modules inclusion into the process, were tested.

### Error analysis and statistics

Error analyses were performed to compare the volitional EMG envelopes, as obtained after extraction from the synthesized data to those obtained directly from the volitional signal. The global error was expressed by the Root Mean Square Error (RMSE), as follows:

RMSE=1N∑1N(CVEMGi−MVEMGi)2⋅100PeakAmpMVEMG     (2)
 MathType@MTEF@5@5@+=feaafiart1ev1aaatCvAUfKttLearuWrP9MDH5MBPbIqV92AaeXatLxBI9gBaebbnrfifHhDYfgasaacH8akY=wiFfYdH8Gipec8Eeeu0xXdbba9frFj0=OqFfea0dXdd9vqai=hGuQ8kuc9pgc9s8qqaq=dirpe0xb9q8qiLsFr0=vr0=vr0dc8meaabaqaciaacaGaaeqabaqabeGadaaakeaacqWGsbGucqWGnbqtcqWGtbWucqWGfbqrcqGH9aqpdaWcaaqaaiabigdaXaqaaiabd6eaobaadaGcaaqaamaaqahabaGaeiikaGIaem4qamKaemOvayLaemyrauKaemyta0Kaem4raC0aaSbaaSqaaiabdMgaPbqabaGccqGHsislcqWGnbqtcqWGwbGvcqWGfbqrcqWGnbqtcqWGhbWrdaWgaaWcbaGaemyAaKgabeaakiabcMcaPmaaCaaaleqabaGaeGOmaidaaaqaaiabigdaXaqaaiabd6eaobqdcqGHris5aaWcbeaakiabgwSixpaalaaabaGaeGymaeJaeGimaaJaeGimaadabaGaemiuaaLaemyzauMaemyyaeMaem4AaSMaemyqaeKaemyBa0MaemiCaa3aaSbaaSqaaiabd2eanjabdAfawjabdweafjabd2eanjabdEeahbqabaaaaOGaaCzcaiaaxMaadaqadaqaaiabikdaYaGaayjkaiaawMcaaaaa@62AD@

where: N is the number of samples, *CVEMG*_*i *_is the i-th sample of the Calculated Volitional EMG (as obtained from the hybrid signal), *MVEMG*_*i *_is the i-th sample of the Measured Volitional EMG (as obtained from the database nominal signal), and *PeakAmp*_*MVEMG *_is the mean peak amplitude of the measured EMG.

This definition enabled a comparative analysis of errors, without being affected by their changing amplitudes.

The local error was expressed by the Mean Amplitude Error (MAE), defined as:

MAE=mean(abs(PeakAMPCVEMG_k−PeakAMPMVEMG_k))mean(PeakAMPCVEMG_k)⋅100     (3)
 MathType@MTEF@5@5@+=feaafiart1ev1aaatCvAUfKttLearuWrP9MDH5MBPbIqV92AaeXatLxBI9gBamXvP5wqSXMqHnxAJn0BKvguHDwzZbqegyvzYrwyUfgarqqtubsr4rNCHbGeaGqiA8vkIkVAFgIELiFeLkFeLk=iY=Hhbbf9v8qqaqFr0xc9pk0xbba9q8WqFfeaY=biLkVcLq=JHqVepeea0=as0db9vqpepesP0xe9Fve9Fve9GapdbaqaaeGacaGaaiaabeqaamqadiabaaGcbaGaemyta0KaemyqaeKaemyrauKaeyypa0ZaaSaaaeaacqWGTbqBcqWGLbqzcqWGHbqycqWGUbGBcqGGOaakcqWGHbqycqWGIbGycqWGZbWCcqGGOaakcqWGqbaucqWGLbqzcqWGHbqycqWGRbWAcqWGbbqqcqWGnbqtcqWGqbaudaWgaaWcbaGaem4qamKaemOvayLaemyrauKaemyta0Kaem4raCKaei4xa8Laem4AaSgabeaakiabgkHiTiabdcfaqjabdwgaLjabdggaHjabdUgaRjabdgeabjabd2eanjabdcfaqnaaBaaaleaacqWGnbqtcqWGwbGvcqWGfbqrcqWGnbqtcqWGhbWrcqGGFbWxcqWGRbWAaeqaaOGaeiykaKIaeiykaKcabaGaemyBa0MaemyzauMaemyyaeMaemOBa4MaeiikaGIaemiuaaLaemyzauMaemyyaeMaem4AaSMaemyqaeKaemyta0Kaemiuaa1aaSbaaSqaaiabdoeadjabdAfawjabdweafjabd2eanjabdEeahjabc+faFjabdUgaRbqabaGccqGGPaqkaaGaeyyXICTaeGymaeJaeGimaaJaeGimaaJaaCzcaiaaxMaadaqadaqaaiabiodaZaGaayjkaiaawMcaaaaa@91BE@

Where: *k *is the gait cycle number, *PeakAMP*_*CVEMG_K *_is the peak amplitude of the Calculated Volitional EMG in cycle *k*, and *PeakAMP*_*MVEG_K *_is the peak amplitude of the Measured Volitional EMG in cycle *k*.

Both RMSE and MAE were calculated for each of the database files using all the EMG processing schemes. ANOVA statistics (α < 0.05) were used to determine statistical differences in the schemes' RMSE and MAE values.

## Results

**Figure 4:**
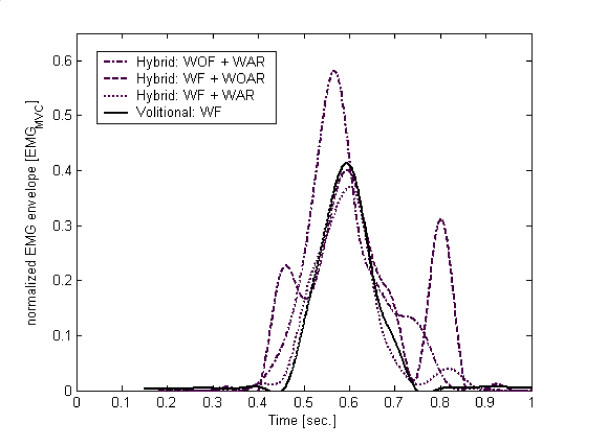
An Illustration of typical volitional EMG envelopes that resulted from processing the synthesized signal, using three different processing schemes. Index: WF – with comb-filter filtering, WOF – without comb-filter filtering, WAR – with artifact suppressor by a blocking time-window, WOAR – without artifact suppressor by a blocking time-window

Figure 4 illustrates typical volitional EMG envelopes that resulted from processing the synthesized signal, using three different processing schemes. The naked eye inspection reveals that the processing scheme including comb-filtering and artifact blocking window gave the closest result to the original volitional EMG signal. Tables 2 and 3 summarize the results of the RMSE and MAE values of the tested subjects and for the implemented processing schemes. The results indicate that the complete process, which included all blocks, consistently yielded low mean RMSE and MAE values compared to the other tested processes.

**Table 2:** Summary of the Normalized Root Mean Square Error (NRMSE) values of the tested subjects for the implemented processing schemes

Processing Module (s)			normalized RMSE of EMG (NRMSE) [%]					
Peaks envelope	Comb filter	Artifact blocking	1 (n = 35)	2 (n = 30)	3 (n = 30)	4 (n = 30)	5 (n = 30)	Mean

-	-		5.35 (2.76)	11.12 (2.65)	8.30 (4.08)	10.37 (3.40)	7.78 (6.13)	8.58
-		-	15.28 (8.12)	9.55 (7.25)	24.18 (11.42)	12.76 (4.94)	29.89 (16.31)	18.33
-			9.51(2.28)	15.74 (1.26)	10.75 (4.57)	13.52 (2.59)	9.61 (3.98)	11.82
	-		7.78 (3.58)	7.75 (5.99)	7.89 (4.52)	8.84 (5.49)	32.66 (17.91)	12.98
		-	18.02 (9.85)	10.52 (7.15)	28.65 (14.69)	14.97 (5.29)	31.57 (17.91)	20.74
			* 1.21(0.87)	* 3.05 (1.54)	* 2.96 (2.56)	* 1.80 (1.21)	* 3.85 (3.32)	2.57

* Significantly lower than all items in same column (α < 0.05)

**Table 3:** Summary of the Mean Amplitude Error (MAE) values of the tested subjects for the implemented processing schemes

Processing Module (s)			Mean Amplitude Error (MAE) [%]				
Peaks envelope	Comb filter	Artifact blocking	1 (n = 35)	2 (n = 30)	3 (n = 30)	4 (n = 30)	5 (n = 30)

-	-		64.81 (8.71)	41.25 (5.98)	33.48 (14.10)	51.97 (6.92)	30.16 (14.31)
-		-	49.96 (54.23)	39.63 (34.21)	128.09 (75.66)	34.51 (24.72)	161.78 (107.28)
-			66.20 (7.64)	43.97 (6.84)	32.15 (12.59)	56.02 (7.23)	41.47 (12.56)
	-		7.94 (7.42)	28.32 (19.78)	34.51 (25.12)	31.05 (14.40)	96.43 (47.88)
		-	73.54 (37.44)	48.11 (37.73)	185.06 (118.35)	52.77 (27.82)	177.77 (104.31)
			8.13 (6.18)	* 7.69 (6.10)	17.98 (15.72)	* 7.58 (5.64)	* 10.46 (8.21)

* Significantly lower than all items in same column (α < 0.05)

The RMSE values for the complete processing scheme, i.e., including all three "flexible" blocks, ranged between 1.21(0.87) and 3.85(3.32), lower compared to every other tested processing scheme. The highest RMSE errors were observed in those processes that did not include the Artifact Blocking Window module and their value ranged between 9.55(7.25) and 31.57(17.91). This points out to the importance of that module for the successful computation of the volitional EMG.

The MAE analyses results were not so conclusive. In 4 out of 5 subjects, the complete processing scheme yielded lower values, between 7.69(6.10) and 17.98(15.72), compared to every other tested processing scheme. In 3 subjects that decrease was significant. In one subject the process which included only two out of three "flexible" blocks, i.e., peak envelope and artifact blocking window, yielded lower (= 7.94) MAE values, compared to the complete process results (8.13). However, this difference was not significant. Similar to the RMSE indication, the MAE values also revealed high errors in processing schemes that did not include the artifact blocking window, with values ranging between 34.51(24.72) to 185.06(118.35)), thus providing further indication to the importance of this block.

## Discussion

The EMG signal provides useful information about the muscle force developed under volitional muscle contraction. In the time domain, the rectified EMG envelope has been widely used for various applications, ranging from simple On/Off activation trigger [9] to muscle force estimation (e.g. [1,26,27]).

In cases of hybrid activation, i.e., when ES is being used to augment volitional muscle activation, the rectified EMG can be used either as a control signal [4-6,14,16], or as a muscle force estimator [11]. In this mode of activation the volitional and ES-induced components of the EMG mix up together, and extraction of the volitional component from the raw EMG signal is required for monitoring and control purposes, necessitating multi-step processing procedures. Methods to extract the volitional component out of the raw EMG signal during hybrid activation have been developed (e.g., [6,12,16]). Few of these reports validate the success in extracting the volitional EMG. To accomplish that, the hybrid EMG signal was emulated by adding up together its volitional and induced components [7,16].

In these latter studies muscle was under static contraction and the EMG signals were represented mathematically (i.e., by an explicit mathematical equation); thus the extraction process necessitated complex processing procedures such as filtration with adaptive filters [16], or Gram-Schmidt prediction error filters [7].

Several methods were used for evaluation of the success of extraction of the volitional EMG. The basic one was by visual inspection of the approximated signal which, despite not providing information on the processing method accuracy, can tell if the signal can be successfully utilized for control purposes [4-6,9,14].

More advanced methods utilized mathematical parameters to score the processing success. Yoem et al. [7] used three evaluation criteria: (a) visual inspection of the power spectra of the signals; (b) comparison of the signals' RMS values; and (c) 'false-positive' parameter that calculated the number of times in which the extracted signal peak amplitude is higher than the maximum value of the pure original EMG ingredient. The obtained parameters' values, which indicated: good visual matching, RMS value close to 1, and small 'false-positive' value, enabled the authors to conclude that a 6^th ^order Gram-Schmidt prediction error filter successfully preserves the original volitional EMG signal.

Sennels et al. [16] illustrated various quantitative tools to test several configurations of adaptive filters. They showed, that for simulated data the adaptive filters they used were relatively insensitive to variations in the muscle responses; however for real-data, it was better to use adaptive filter with a large number of elements. Their results led them to the conclusion that a 6-element adaptive filer can successfully eliminate the ES share from the hybrid EMG signal.

The methodology developed in the present study was different in two respects: (a) each of the activation components was the result of dynamic, rather than static contraction, and (b) the signals were represented numerically, by means of their sampled actual values, obtained from repetitive dynamic contraction data simulating typical gait-like activity of the TA muscle during a swing phase. The advantage of this approach is that it better reflects real-life situation, whereby signals are normally dynamic, rather than static and they may not always be represented mathematically.

Additionally, since we were interested in the EMG envelope of the volitional component rather than in its raw signal, the resulting processing scheme turned to be much simpler than the one developed in the abovementioned previous works. This concept is different from other works, in which it is first attempted to obtain the raw volitional signal, and then apply standard processing routines to derive its envelope [4-6,8,12,16,20]. The idea here is that since the envelope pattern is smoother and well-defined compared to the raw EMG, it can be recovered more accurately and with less complexity, compared to the traditional methods. Those advantages are of great relevance when using the procedure for real-time applications.

Basically, the signal processing scheme relies on commonly used routines, involving 'rigid' and 'flexible' modules. The 'rigid' modules, which include high-pass filtration, signal rectification, and low-pass filtration, are commonly used for EMG envelope calculations (e.g.: [1,20]); and the 'flexible' modules are commonly found in the context of ES artifacts removal [6,12,15,17,21]. The role of each module in the process is well defined: The "Artifact Blocking Window" module suppresses the ES artifact (e.g.: [6,12], [15]). The Comb-filter filtration module further cleans the signal from the ES component [12]. The Rectified signal peak envelope module provides a rough representation of the EMG envelope, and reconstructs its pattern in regions where the signal has been chopped out for artifact suppression (e.g., in the blocked regions). A processing scheme that combines together these modules is not found in literature.

Several works dealt with the ES artifact with utilization of only one of the above modules; mostly Comb-filter filtration or Artifact Blocking Window [12,20,21]. Frigo et al. [12] have used both Comb-filter filtration and Artifact Blocking Window modules, and reported on improved elimination of ES component.

Our work has shown that all three 'flexible' modules are necessary for the appropriate elimination of the ES component from the overall signal. However, when only the Comb-filter or Artifact Blocking Window module takes part in the process, we demonstrated that inclusion of the Rectified peak envelope module was not necessary and could introduce substantial errors. This is because the Rectified Peak Envelope module is only effective when the signal is completely cleaned from its ES component.

When the Comb-filter filtration and Artifact Blocking Window were joined together in the process, the rectified signal did not have any substantial ES remainders and the envelope reconstruction was successful. However, when only one of the above modules was used, there were still some ES residuals [12] that led to an erroneous envelope reconstruction.

It should be noted, though, that in the Rectified signal peak envelope module, a complete period of the signal is required in order to reconstruct its blocked regions. This introduces a time delay of one period, thus preventing real-time application of the Rectified signal peaks envelope module. For real-time applications, the literature has suggested several solutions to emulate the operation of this module, the most common being a blocking window with an average signal value, or a holder that retains the pre-blocking last sample value of the signal [8,20,21]. This allows for the reconstruction of the missing samples, thus providing its real-time envelope approximation.

To compare between the calculated volitional EMG-envelope (i.e., from synthetic database) and the actual one (from volitional only tests), we used two parameters, RMSE and MAE, for the large and small scale comparisons, respectively.

The RMSE expresses the mean difference between two signals, and reflects their general similarity. This parameter is widely used in literature (e.g: [7,22]).

The MAE parameter expresses the difference between the approximated and actual signal amplitudes. Assuming that the signals do not have any difference in phase and shape, the difference in amplitudes represents the local error. Since many applications use the value of the EMG envelope as a control signal [4-6,8,12], MAE becomes important and knowledge of the envelope approximation error is required for system design. A similar MAE analysis is not found in literature, probably since all the related works dealt with recovery of the raw volitional-EMG signal and not of its envelope (e.g., [7,20,21,23]).

The suggested method in our work provides rational evaluators for the performance of the various schemas. These, however, are not the only possible evaluators. For instance, Sennels et al [16] used other evaluators to test the success of their methods. The first evaluator was used on simulated data, and examined the ratio between the input signal to noise ratio (SNR) and the output SNR; the second evaluator was used on real-data, and examined the power reduction of the input and output signals. Thus, further works should enable unbiased comparisons between current and earlier works.

It is noticeable that the variance of the MAE, and RMSE values between the subjects was high. Several reasons can lead to such behavior; e.g.: differences in anatomy, tissue structure, muscle fatigue, etc., which have an effect on the subject's ES pattern, and therefore on the schema performance. Nevertheless, for the selected schema, the obtained low values of RMSE and MAE (RMSE: 1.21 – 3.85%, MAE: 7.58 – 10.46%) in all the subjects but one, indicate the high accuracy of the processing method, and point out that the preferred computational scheme should include all modules.

A major assumption was made in this work, according to which the hybrid EMG can be represented by the superposition of the volitional only and induced only EMG signals. This assumption relied on a preliminary work, which verified that a superimposed signal has the characteristics of an actual hybrid signal; thus, the superimposed hybrid signal has a typical spike and M-wave which can be related to the ES component, together with a stochastic-like signal resulting from the volitional component. In addition, since the origins of the superimposed signal are known, we can establish an unbiased comparison between the various signal-processing schemes and their ability to satisfactorily resolve the hybrid signal into its components.

When a preferred signal-processing scheme is obtained, we can apply it to an *in-vivo *hybrid EMG signal with high certainty that the process outcome well-describes the hybrid signal origins.

Another assumption was that the simulated dynamic motion torque reflects the actual gait profile. This assumption should be further examined due to the fact that the actual gait motion is non-isometric, and is influenced by body kinematics that could in turn be reflected on the volitional and induced EMG signals. Nevertheless, the generality of the selected schema enables its implementation to more realistic gait-like signals without any noticeable changes.

## Summary and conclusion

This work defined a computational scheme to extract, from the overall dynamic EMG signal, the volitional EMG envelope, to serve as a basic signal for control and force evaluation. For this purpose, a "synthetic" database was created from *in-vivo *experiments to emulate hybrid EMG signals, including the volitional and induced components. The database was used to evaluate the performance of six different signal processing schemes. Performance was evaluated by means of two measures that examined the process success both from the local and in the global aspects: RMSE and MAE, respectively. The results indicated that the processing scheme that included seven modules led to an excellent approximation of the volitional EMG envelope after extraction from the hybrid signal. Moreover, it underlined the importance of Artifact Blocking Window module in the process. The results of this work have direct implications on the development of hybrid muscle activation rehabilitation systems. Future work should focus on the implementation of the algorithm in actual rehabilitation systems and on the evaluation of its performance in real-time conditions.

## Authors' contributions

EL participated in the design of the study, carried out the experiments, data analysis and drafted the manuscript. EI participated in the design of the study, especially the clinical aspects. JM participated in the design of the study, drafted the manuscript, and supervised the research. All authors read and approved the final manuscript.
